# Anticoagulant Impact on Clinical Outcomes of Pulmonary Embolism Compared With Thrombolytic Therapy; Meta‐Analysis

**DOI:** 10.1002/clc.70016

**Published:** 2024-09-12

**Authors:** Yang Su, Dongmei Zou, Yi Liu, Chaoqun Wen, Xialing Zhang

**Affiliations:** ^1^ Department of Cardiovascular surgery, Changde Hospital, Xiangya School of Medicine Central South University (The First People's Hospital of Changde City) Changde Hunan China

**Keywords:** anticoagulant, CDT, pulmonary embolism, thrombolytic therapy

## Abstract

**Background:**

Pulmonary embolism (PE) is a critical condition requiring effective management strategies. Several options are available, including thrombolytic therapy and anticoagulants.

**Objectives:**

To assess the impact of thrombolytic therapy either combined with anticoagulant (AC) or alone versus AC alone on mortality, recurrence, clinical deterioration, bleeding, and hospital stay.

**Method:**

This study included 25 previously published studies from 1990 to 2023, with a total of 12 836 participants. Dichotomous and continuous analysis models were used to evaluate outcomes, with heterogeneity and publication bias tests applied. A random model was used for data analysis. Several databases were searched for the identification and inclusion of studies, such as Ovid, PubMed, Cochrane Library, Google Scholar, and Embase.

**Results:**

For sub‐massive PE, CDT plus AC significantly reduced in‐hospital, 30‐day, and 12‐month mortality compared to AC alone, odds ratio (OR) of −0.99 (95% CI [−1.32 to −0.66]), with increased major bleeding risk but no difference in minor bleeding or hospital stay, OR = 0.46, 95% CI [−0.03 to 0.96]). For acute intermediate PE, systemic thrombolytic therapy did not affect all‐cause or in‐hospital mortality but increased minor bleeding, reduced recurrent PE, and prevented clinical deterioration. The heterogeneity of different models in the current study varied from 0% to 37.9%.

**Conclusion:**

The addition of CDT to AC improves mortality outcomes for sub‐massive PE but raises the risk of major bleeding. Systemic thrombolytic therapy reduces recurrence and clinical decline in acute intermediate PE despite increasing minor bleeding. Individualized patient assessment is essential for optimizing PE management strategies.

## Graphical abstract

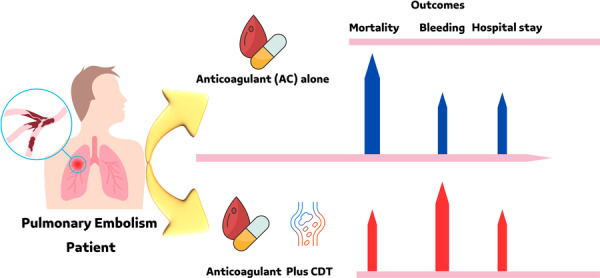

## 1 Introduction

Pulmonary embolism (PE) is a critical medical condition characterized by the sudden blockage of a major blood vessel in the lung, usually caused by a blood clot. PE is a significant cause of morbidity and mortality worldwide, and its management is crucial for improving patient outcomes [1]. The clinical presentation of PE can vary widely, ranging from asymptomatic cases to sudden death. The severity of PE is often categorized based on the patient's hemodynamic stability and the extent of the clot burden, leading to classifications such as sub‐massive and massive PE [2].

In sub‐massive pulmonary embolism (sPE), patients are hemodynamically stable but exhibit right ventricular dysfunction or myocardial necrosis. Acute intermediate‐risk PE, on the other hand, includes patients with signs of right ventricular dysfunction but without hypotension [3, 4].

Exclusion or identification of right ventricular strain in hemodynamically stable patients with PE is a component of the risk stratification that directs additional therapy (“intermediate care,” normal ward, outpatient treatment). Echocardiography can also identify significant concomitant conditions and help identify symptoms of a preexisting chronic cor pulmonale. Thus, even in patients with stable PE, echocardiography needs to be a standard technique, even though current recommendations do not require it [5].

The management of PE has evolved significantly over the years, with anticoagulation therapy being the cornerstone of treatment. Anticoagulants such as heparin and warfarin help prevent further clot formation and facilitate clot resolution [2]. However, anticoagulation alone may not be sufficient for all patients, particularly those with extensive clot burden or right ventricular dysfunction. In such cases, more aggressive interventions, including thrombolysis and catheter‐directed therapies, may be considered [6]. Although prolonged treatment is related to a decreased chance of recurrence, it is also associated with a higher risk of bleeding. This is the reason why the length of oral anticoagulation is a more contentious argument. Oral anticoagulant medication should be administered for a minimum of 3 months to every patient. The selection of candidates for prolonged oral anticoagulation should be based on the stratification of the risk of recurrence, which has been estimated to be approximately 2.5% per year after PE associated with transient risk factors and 4.5% per year after PE occurring in the absence of transient risk factors or in patients with cancer or thrombophilia. This information ought to be taken into consideration when selecting candidates for prolonged oral anticoagulation [7, 8].

Catheter‐directed thrombolysis (CDT) involves the use of a catheter to deliver thrombolytic agents directly to the site of the clot. This approach aims to enhance clot dissolution while minimizing systemic exposure to thrombolytics, thereby reducing the risk of major bleeding [9, 10]. CDT has gained attention as a potential therapy for sPE, where the goal is to reduce right ventricular strain and improve hemodynamic outcomes without the high bleeding risks associated with systemic thrombolysis [11, 12].

Systemic thrombolytic therapy, which involves the intravenous administration of thrombolytic agents, has been traditionally reserved for massive PE with hemodynamic instability [13]. However, its role in the treatment of acute intermediate‐risk PE remains controversial. While systemic thrombolysis can effectively dissolve clots and improve pulmonary perfusion, it is also associated with significant bleeding complications. Thus, the balance between efficacy and safety is a critical consideration in its use [14].

The decision‐making process for the management of PE is complex, involving the assessment of patient‐specific factors such as the severity of the PE, comorbid conditions, and the risk of bleeding [15]. The heterogeneity of PE presentations and the varying responses to treatment further complicate this process. Additionally, the optimal therapeutic approach for sub‐massive and intermediate‐risk PE continues to be debated, with ongoing research aiming to delineate the most effective and safest strategies [15, 16, 17].

Given the significant morbidity and mortality associated with PE, there is a pressing need for evidence‐based guidelines to inform clinical practice. Meta‐analyses offer a robust method for synthesizing data from multiple studies, providing a comprehensive overview of treatment efficacy and safety.

## 2 Objective

The objective of this meta‐analysis is to assess the impact of using thrombolysis alone or combined with anticoagulant (AC) on mortality, bleeding, recurrence, clinical deterioration, and hospital stay in patients with PE. This analysis will provide critical insights to guide clinical decision‐making and optimize patient outcomes in the management of PE.

## 3 Methods

### 3.1 Study Design

**Figure 1:**
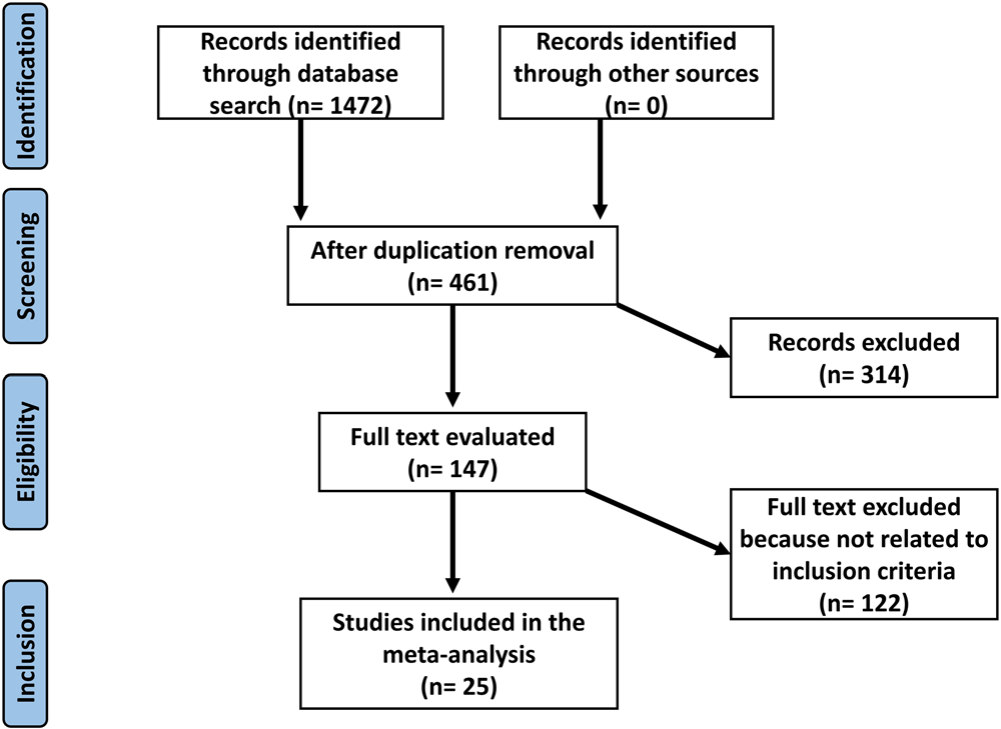
Schematic illustration of the recursion process of analyzed studies.

This meta‐analysis was conducted to evaluate the efficacy and safety of different treatment strategies for managing PE. Adhering to an epidemiological declaration, we followed a structured protocol to ensure rigorous analysis. Data were gathered from several scientific databases, including PubMed, Cochrane Library, OVID, and Embase, and analyzed according to the specified inclusion criteria [18]. The entire process is illustrated in Figure 1.

### 3.2 Eligibility and Inclusion

The primary focus was to assess the impact of CDT plus anticoagulation versus AC alone, and systemic thrombolytic therapy versus AC. Articles included in the study specifically examined these treatment modalities and their outcomes related to mortality, bleeding events, recurrence, hospital stay, and clinical deterioration.

### 3.3 Inclusion Criteria

– Only human‐based clinical trials published up until December 2023 were included.
– Studies must involve patients with PE receiving either CDT plus AC or systemic thrombolytic therapy versus AC alone.
– Trials comparing the effects of these interventions on mortality, bleeding events, recurrence, hospital stay, and clinical deterioration were required.

### 3.4 Exclusion Criteria

– Articles that did not present continuous model analysis data using mean and standard deviation (SD) for various outcomes were excluded. Also, studies did not indicate accurately if participants received related medication that could affect the results also excluded.
– Studies published as book chapters, letters, or review articles were not considered as they have a weak methodology and no detailed intervention.

### 3.5 Identification

Our search strategy was implemented up until December 2023 using a combination of terms related to PE, CDT, systemic thrombolysis, anticoagulation, mortality, bleeding, recurrence, and clinical outcomes. The search protocol was defined according to the PICOS framework as follows:

– P (Population): Patients with PE.
– I (Intervention/exposure): CDT plus anticoagulation, and systemic thrombolytic therapy.
– C (Comparison): Anticoagulation alone.
– O (Outcomes): Mortality, bleeding events, recurrence, and clinical deterioration.
– S (Study design): Randomized clinical trials.

The search encompassed PubMed, Cochrane Library, Embase, OVID, and Google Scholar databases. Articles were screened based on titles and abstracts to exclude those not evaluating the specified interventions, and the relevant studies were imported into a reference management program.

### 3.6 Screening

Data extraction involved filtering based on specific criteria such as the first author's surname, year of publication, country, study type, study duration, demographic information, clinical and treatment characteristics, number of subjects, methods, information sources, and outcomes [19]. Each selected study's methodological quality was assessed to identify potential biases. Reviewer manager software was used to evaluate the risk of bias for each included study, categorizing them as low, medium, or high risk. Several databases were searched, such as Ovid, PubMed, Cochrane Library, Google Scholar, and Embase using keywords such as PE, sub‐massive, anticoagulant, intermediate PE, and thrombolytic agents.

### 3.7 Statistical Analysis

In this meta‐analysis, we calculated the mean difference (MD) and estimated odd ratio (OR) with a 95% confidence interval (CI) using a random‐effects model, as there was variability among the included studies. The use of a random model is considered due to the lack of high similarity between included studies. Results were presented as forest plots, illustrating the CI of each group and the overall direction of the effects across studies. Group and subgroup analyses were conducted to examine the overall results and the impact of specific factors shared by smaller subsets of studies.

The heterogeneity among studies was quantified using the *I*² index, which ranges from 0% to 100%, indicating low, moderate, or high levels of heterogeneity. The *τ*² statistic was calculated using the restricted maximum likelihood estimator. Jamovi software was used for statistical analyses, including assessing publication bias through Begg's and Egger's tests and visual inspection of funnel plots.

## 4 Results

**Figure 2:**
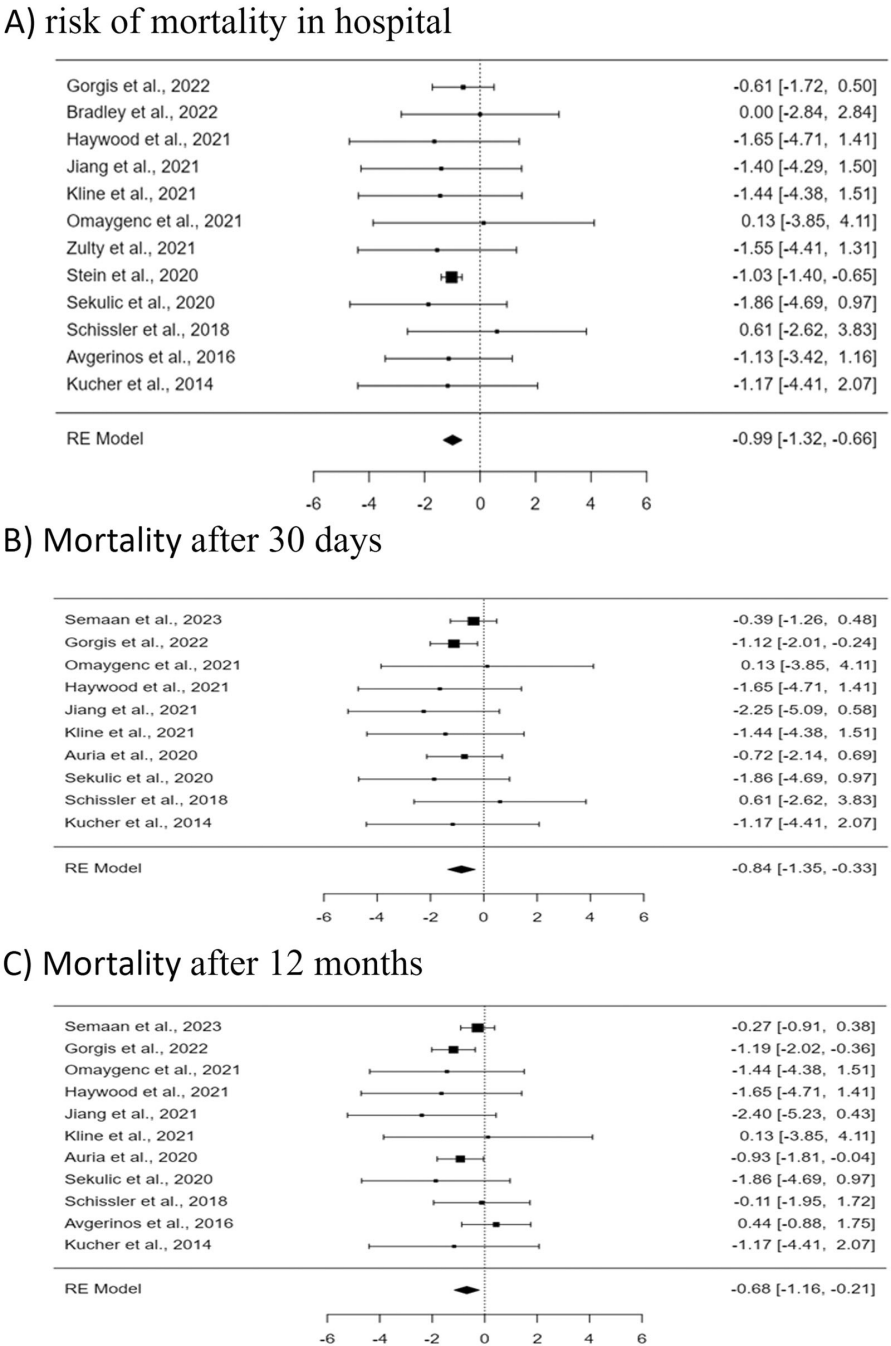
Forest plot showing the impact of CDT plus AC therapy compared with AC alone on risk of mortality in hospital (A), after 30 days (B), and after 12 months (C).

**Figure 3:**
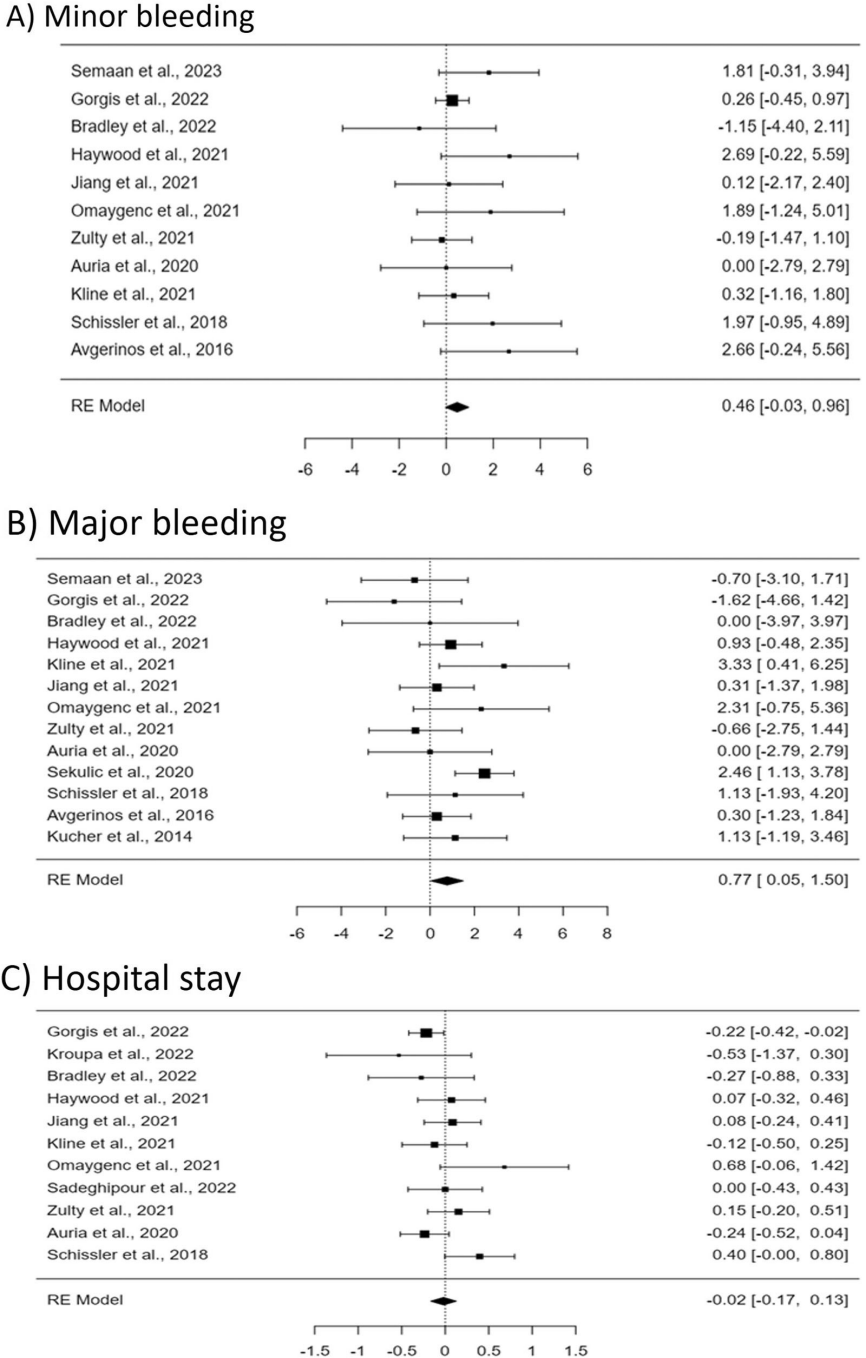
Forest plot showing the impact of CDT plus AC therapy compared with AC alone on risk of bleeding for both minor (A), major bleeding (B), and hospital stay (C).

**Figure 4:**
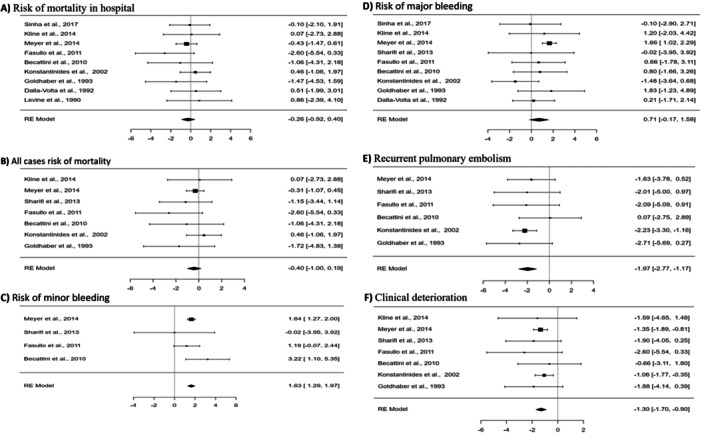
Forest plot showing the impact of systemic thrombolytic therapy compared with AC alone on risk of mortality in hospital (A), all causes mortality (B), risk of minor (C) and major bleeding (D), risk of recurrent pulmonary embolism (E), and clinical deterioration (F).

Findings of this study regarding the impact of different interventions for the management of PE (sub‐massive and acute intermediate PE). Studies comparing CDT plus AC versus AC for the management of submissive pulmonary embolism are analyzed and results are expressed in Table 1 and Figures 2 and 3. While results of the analysis of studies comparing systemic thrombolytic versus AC for the management of acute intermediate PE are expressed in Table 1 and Figure 4. The current study included 25 previously published studies from 1990 to 2023 with a total number of participants = 12 836; from them, 9261 participants received AC alone and the rest of the population received either AC plus CDT (3125) or systemic thrombolytic (9261) [20, 21, 22, 23, 24, 25, 26, 27, 28, 29, 30, 31, 32, 33, 34, 35, 36, 37, 38, 39, 40, 41, 42, 43, 44].

**Table 1:** Study characteristics.

Study	Year	Thrombolytic	Anticoagulant	Total participant	Included patients
Semaan et al. [20]	2023	235	235	470	Sub‐massive pulmonary embolism
Bradley et al. [23]	2022	21	21	42	Sub‐massive pulmonary embolism
Gorgis et al. [22]	2022	192	192	384	Sub‐massive pulmonary embolism
Kroupa et al. [21]	2022	12	11	23	Sub‐massive pulmonary embolism
Kline et al. [26]	2021	40	90	130	Sub‐massive pulmonary embolism
Haywood et al. [28]	2021	51	51	102	Sub‐massive pulmonary embolism
Jiang et al. [27]	2021	47	158	205	Sub‐massive pulmonary embolism
Omaygenc et al. [25]	2021	14	16	30	Sub‐massive pulmonary embolism
Zulty et al. [24]	2021	37	177	214	Sub‐massive pulmonary embolism
Auria et al. [31]	2020	99	99	198	Sub‐massive pulmonary embolism
Stein et al. [29]	2020	1260	6910	8170	Sub‐massive pulmonary embolism
Sekulic et al. [30]	2020	24	227	251	Sub‐massive pulmonary embolism
Schissler et al. [32]	2018	65	39	104	Sub‐massive pulmonary embolism
Sinha et al. [33]	2017	45	41	86	Acute intermediate pulmonary embolism
Avgerinos et al. [34]	2016	64	64	128	Sub‐massive pulmonary embolism
Kucher et al. [36]	2014	30	29	59	Sub‐massive pulmonary embolism
Meyer et al. [35]	2014	506	499	1005	Acute intermediate pulmonary embolism
Kline et al. [37]	2014	40	43	83	Acute intermediate pulmonary embolism
Sharifi et al. [38]	2013	61	60	121	Acute intermediate pulmonary embolism
Fasullo et al. [39]	2011	37	35	72	Acute intermediate pulmonary embolism
Becattini et al. [40]	2010	28	30	58	Acute intermediate pulmonary embolism
Konstantinides et al. [41]	2002	118	138	256	Acute intermediate pulmonary embolism
Goldhaber et al. [42]	1993	46	55	101	Acute intermediate pulmonary embolism
Dalla‐Volta et al. [43]	1992	20	16	36	Acute intermediate pulmonary embolism
Levine et al. [44]	1990	33	25	58	Acute intermediate pulmonary embolism

### 4.1 CDT Plus AC Versus AC Alone

For the management of submissive pulmonary embolism, the addition of CDT to anticoagulation was associated with significant improvements in mortality outcomes compared to AC alone. In an analysis of 12 studies, in‐hospital mortality was significantly lower for the CDT plus AC group, with a *p*‐value of less than 0.0001, and an estimated OR of −0.99 (95% CI [−1.32 to −0.66]). The heterogeneity of this model was negligible (*I*² = 0.0%), indicating consistency in outcomes across the studies. Similarly, the analysis of 10 studies showed that the 30‐day mortality was significantly lower in the CDT plus AC group (*p* = 0.001, OR = −0.84, 95% CI [−1.35 to −0.33]), also with no observed heterogeneity (*I*² = 0.0%). Additionally, 11 studies indicated a significant reduction in 12‐month mortality for the CDT plus AC group compared to the AC alone group, with a *p*‐value of 0.004 and an OR of −0.68 (95% CI [−1.16 to −0.21]). The heterogeneity was low (*I*² = 16.1%), suggesting relatively consistent outcomes.

In terms of bleeding events, the analysis of 11 studies revealed that there was no significant difference in minor bleeding incidents between the CDT plus AC group and the AC alone group (*p* = 0.0639, OR = 0.46, 95% CI [−0.03 to 0.96]), with negligible heterogeneity (*I*² = 0.0008%). However, 13 studies indicated a significant increase in major bleeding in the CDT plus AC group (*p* = 0.0369, OR = 0.77, 95% CI [0.05 to 1.50]), with moderate heterogeneity (*I*² = 30.8%). These results highlight an increased risk of major bleeding associated with the addition of CDT to AC.

Regarding the length of hospital stay, 11 studies were analyzed using a continuous analysis model. The findings indicated no significant difference in hospital stay duration between the CDT plus AC group and the AC alone group (*p* = 0.8017, standardized mean difference [SMD] = −0.02, 95% CI [−0.17 to 0.13]). The heterogeneity was moderate (*I*² = 37.9%), suggesting some variation in outcomes across the studies.

### 4.2 Systemic Thrombolytic Versus AC Alone

For the management of acute intermediate PE, systemic thrombolytic therapy was compared to AC alone. Analyzing seven studies, the results showed no significant difference in all‐cause mortality between the systemic thrombolytic therapy group and the AC group (*p* = 0.1851, OR = −0.40, 95% CI [−1.00 to 0.19]), with negligible heterogeneity (*I*² = 0.0%). Additionally, nine studies indicated no significant difference in in‐hospital mortality between the two groups (*p* = 0.4336, OR = −0.26, 95% CI [−0.92 to 0.40]), with negligible heterogeneity (*I*² = 0.0%).

In terms of bleeding events, four studies revealed a significant increase in minor bleeding incidents with systemic thrombolytic therapy compared to AC alone (*p* < 0.0001, OR = 1.63, 95% CI [1.29 to 1.97]), with negligible heterogeneity (*I*² = 0.0002%). Conversely, nine studies showed no significant difference in major bleeding between the two groups (*p* = 0.1142, OR = 0.71, 95% CI [−0.17 to 1.58]), although there was moderate heterogeneity (*I*² = 33.5%).

The analysis also examined the recurrence of PE. Six studies indicated a significant reduction in recurrent PE with systemic thrombolytic therapy compared to AC alone (*p* < 0.0001, OR = −1.97, 95% CI [−2.77 to −1.17]), with negligible heterogeneity (*I*² = 0.0%). Moreover, seven studies demonstrated a significant reduction in clinical deterioration with systemic thrombolytic therapy compared to AC alone (*p* < 0.0001, OR = −1.30, 95% CI [−1.70 to −0.90]), also with negligible heterogeneity (*I*² = 0.0%).

These detailed findings provide valuable insights into the comparative effectiveness and safety of different treatment strategies for managing PE. The data underscore the benefits and risks associated with each treatment approach, guiding clinical decision‐making for optimal patient outcomes.

The study's analysis of publication bias across various outcomes revealed consistent findings, with minimal indications of publication bias. For 30‐day mortality, neither the rank correlation nor the regression test indicated funnel plot asymmetry (*p* = 0.6007 and *p* = 0.5915, respectively). Similarly, for 12‐month mortality, the tests showed *p*‐values of 0.8793 and 0.4034. In‐hospital mortality had *p*‐values of 0.5452 and 0.8580. Minor bleeding yielded *p*‐values of 0.4454 and 0.1018, while major bleeding had *p*‐values of 0.9524 and 0.5530. For hospital stay, the tests indicated *p*‐values of 0.5423 and 0.3350. Outcomes related to systemic thrombolytic treatment also showed nonsignificant *p*‐values, with all‐cause mortality at 0.2389 and 0.2432, and in‐hospital mortality at 0.7614 and 0.6734. In recurrent PE, the *p*‐values were 1.0000 and 0.5315, and for clinical deterioration, they were 0.7726 and 0.4983. None of the studies showed extreme outliers or overly influential data points, indicating that the results across the studies are likely robust and not significantly impacted by publication bias.

## 5 Discussion

The meta‐analysis of PE management provides significant insights into the comparative effectiveness of different treatment strategies, specifically the addition of CDT to anticoagulation for sPE and the use of systemic thrombolytic therapy versus AC for acute intermediate PE.

For sPE, the combination of CDT with AC was associated with improved mortality outcomes. Previous studies have demonstrated that CDT can rapidly reduce right ventricular afterload and improve right heart function, which is crucial in the management of sPE. The reduction in in‐hospital, 30‐day, and 12‐month mortality rates observed in our analysis aligns with these findings, highlighting the potential benefits of CDT in reducing early and late mortality in patients with sPE. However, the increased risk of major bleeding associated with CDT necessitates careful patient selection and management to mitigate this risk. The lack of significant differences in minor bleeding and hospital stay duration between the CDT plus AC and AC alone groups suggests that while CDT offers survival benefits, it does not substantially impact less severe bleeding complications or hospitalization time.

The study findings are in line with the guidelines, highlighting that while CDT can improve mortality outcomes, it carries a significant risk of major bleeding. This reinforces the cautious approach recommended in guidelines, where CDT is reserved for select patients rather than being applied broadly. In addition, the study's findings that systemic thrombolysis does not significantly reduce mortality but does reduce recurrent PE and clinical deterioration align with the cautious stance of current guidelines. The increased risk of minor bleeding also supports the guideline recommendation to limit systemic thrombolysis to patients at higher risk of adverse outcomes, rather than applying it universally in intermediate‐risk PE.

The lower likelihood of death from any cause in the CDT group may be associated with improved right ventricular function and reduced pulmonary artery pressure. CDT provides numerous advantages compared to AC. First, it efficiently reduces systolic pulmonary artery pressure (SPAP) and mean pulmonary artery pressure (MPAP). Furthermore, when comparing the AC group to the CDT group, it is evident that the CDT group shows significant and swift enhancements in right ventricular function and cardiac index. Furthermore, CDT is more effective in reducing the burden of pulmonary thrombosis compared to AC [21, 25, 36]. The characteristics of a quick drop in pulmonary artery pressure, enhanced right ventricular function, and reduced pulmonary thrombotic load may explain the ability of CDT to lower overall mortality in patients with sPE [45, 46].

Assessing the precise likelihood of significant hemorrhage based on available literature is hindered by various complexities. Various thrombolytic drugs and dose regimes are used in individual research, each with its own distinct dangers. Sample sizes frequently lack sufficient magnitude to evaluate the risk associated with a specific treatment plan. There is inconsistency in the definitions of substantial bleeding across different research. Early studies sometimes reported bleeding issues associated with the vascular access sites used for pulmonary angiography, which often required blood transfusions. The rates of PE diagnostic tests may be lower in modern practice using noninvasive methods [47]. A prior investigation showed the potential hazards of significant bleeding in patients who underwent therapy for PE using a dosage of 100 mg of alteplase at a solitary medical facility. The analysis identified several significant risk factors. These included having undergone major surgery within the past 3 weeks (OR 9.00, 95% CI 1.01−79.99), having an international normalized ratio (INR) greater than 1.7 (OR 13.20, 95% CI 1.54−113.52), weighing less than 100 kg (OR 1.18 for every 10 kg below 100 kg, 95% CI 1.01−1.37), and having at least one of the following characteristics (OR 5.02, 95% CI 1.78−18.55): internal bleeding in the previous 4 weeks, hypertension, acute myocardial infarction, positive stool occult test, presence of an intra‐aortic balloon pump, African American race, gastrointestinal bleeding in the previous 3 months, aortic dissection, acute pancreatitis, cardiopulmonary resuscitation lasting more than 10 min, bilirubin level greater than 3 mg/dL, or dementia [48].

The systemic thrombolytic therapy for acute intermediate PE did not show a significant impact on all‐cause or in‐hospital mortality compared to AC alone. This finding is consistent with previous research indicating that while thrombolytic therapy can be beneficial in high‐risk PE, its advantages in intermediate‐risk PE are less clear. However, our analysis revealed a significant increase in minor bleeding incidents with systemic thrombolytic therapy, which corroborates the known bleeding risks associated with thrombolytics. Interestingly, the significant reduction in recurrent PE and clinical deterioration with systemic thrombolytic therapy suggests that it may offer protective benefits against PE recurrence and subsequent complications. These findings support the notion that systemic thrombolytic therapy may be beneficial in preventing the progression and recurrence of PE, particularly in patients at higher risk for clinical deterioration.

Cao et al. determined that thrombolysis, when used for acute sPE, did not result in a decrease in mortality or recurrent PE, nor did it lead to an increase in bleeding hazards [49]. Nevertheless, their research was constrained by a small sample size of only 594 patients and did not include the most recent clinical trial discoveries. Chatterjee et al. discovered that the use of thrombolytic treatment for acute PE was associated with reduced overall mortality [50]. However, it also carried an increased risk of significant bleeding and brain hemorrhage. Thrombolysis in intermediate‐risk PE patients resulted in a decrease in fatality rates, but it also led to an increase in the occurrence of significant bleeding episodes. Although our meta‐analysis validated their findings on death, it presented contrasting results on the hazards of bleeding. The reason for this difference could be that we did not include data from the ULTIMA trial [36], which used catheter‐directed alteplase, and Goldhaber et al.'s study, which did not provide clear information about significant bleeding events in the subgroup with right‐ventricular hypokinesis [42]. These factors probably influenced the variability in the assessment of significant bleeding incidents.

Overall, these findings underscore the importance of individualized patient assessment and risk stratification in the management of PE. The use of CDT plus AC in sPE offers significant mortality benefits but comes with increased bleeding risks, necessitating a balanced consideration of benefits and risks. Systemic thrombolytic therapy in acute intermediate PE does not significantly reduce mortality but offers protection against recurrence and clinical deterioration, suggesting its potential role in preventing long‐term complications of PE. Future research should focus on refining patient selection criteria and optimizing treatment protocols to enhance the safety and effectiveness of these interventions.

## 6 Limitations

This meta‐analysis has some limitations. There was variability among the included studies regarding design, patient populations, intervention protocols, and outcome measures, which could be resolved using a random‐effects model. Publication bias remains a possibility, especially since studies with negative or inconclusive results are less likely to be published. Inconsistent reporting of outcomes and the focus on short‐term outcomes posed challenges, providing limited information on long‐term effects. In addition, the current study did not discuss the impact of differences in anticoagulant dosages or timing of thrombolysis. Only English‐language studies were included, potentially excluding relevant research in other languages and affecting the comprehensiveness of the findings.

## 7 Conclusion

The meta‐analysis highlights the nuanced benefits and risks associated with different treatment strategies for PE. CDT plus AC significantly improves mortality outcomes for sPE but increases the risk of major bleeding. Systemic thrombolytic therapy does not significantly impact overall mortality for acute intermediate PE but reduces the risk of recurrence and clinical deterioration, despite increasing minor bleeding incidents. These findings underscore the importance of individualized patient assessment and risk stratification in the management of PE.

## Ethics Statement

The authors have nothing to report.

## Conflicts of Interest

The authors declare no conflicts of interest.

## Data Availability

The data that support the findings of this study are available on request from the corresponding author. The data are not publicly available due to privacy or ethical restrictions.
